# Morphological characteristics and functional adaptation of gills in the Meagre, *Argyrosomus regius (Asso, 1801)*

**DOI:** 10.1186/s12917-025-04512-6

**Published:** 2025-02-10

**Authors:** Basma G. Hanafy, Mohamed M.A. Abumandour

**Affiliations:** Department of Anatomy and Embryology, Faculty of Veterinary Medicine, Alexandria University, P.O. 21944, Abis 10th, Alexandria, Egypt

**Keywords:** *Argyrosomus regius (Asso, 1801)*, Gill arch, Gill filaments, Gill rakers, Gill spines, SEM study

## Abstract

A comprehensive morphological description of *Argyrosomus regius (Asso, 1801)* gills was conducted through the gross and scanning electron microscopic examinations in addition to the morphometric analysis of the number, length of the gill rakers and the space between them. The medial surface of the 1st gill arch was differed than that of the other three gill arches. Four gill arche's lateral surfaces contained pear-shaped, circular, and oval-shaped spine groups with varying sizes. The medial surface of the 1st gill arch only had different sizes of pear-shaped and oval groups of spines, while the other gill arches had the same-shaped groups of spines beside the presence of small circular groups. The lateral rakers of the 1st gill arch were long, and only one border carried pointed spines, while its medial rakers were triangular. The rakers of the 2nd, 3rd, and 4th gill arches appeared flask like medially and laterally. There were few folds and tubercles appearing on the primary gill filament surfaces; some folds appeared like the helix on the surface of their parts near the gill arch convex border. This is the first anatomical study on *Argyrosomus regius* gills and we aimed to reveal the unique structural specifications for gill rakers that related to its feeding behavior and we will contribute to a better understanding of its ecological niche and feeding strategies in its natural environment.

## Highlights

• The lateral surface of the four gill arches of *Argyrosomus regius* had spines in the form of pear-shaped and circular groups that differed in size beside the presence of oval-shaped groups.

• The medial surface of *Argyrosomus regius* 1st gill arch only had different sizes of pear-shaped groups and oval groups. While the medial surface of the 2nd, 3rd, and 4th gill arches had the same-shaped groups of spines beside the presence of small circular groups.

• The *Argyrosomus regius* 1st gill arch had long lateral rakers and triangular medial rakers, while the medial and lateral rakers of the other three gill arches were flask like

• The study revealed that *Argyrosomus regius* gill rakers poss unique structural specifications related to its feeding behavior.

## Introduction

*Argyrosomus regius (Asso, 1801)* (Perciformes: Sciaenidae) is a migratory fish distributed in the Black Sea, Mediterranean Sea, and along the Atlantic coasts of Europe and the west coast of Africa. It is present in inshore and shelf waters near the bottom or near the surface in a depth ranged from 15 m to 200 m; it also enters coastal lagoons and estuaries [1]. It provides flesh of low fat even under intensive farming. It may be a good candidate for aquaculture because of its rapid growth, high flesh quality and flavor, and high tolerance to salinity [2].

The gills are considered to be the gas exchange places for respiration [3], and they have a secondary function related to feeding, as the gill rakers and filaments arrangement expresses the fish feeding habits [4–8]. The sizes of the eaten food particles are controlled by the organization and size of the gill rakers; fish with many long rakers are filter feeders, while the fish with few short rakers are carnivorous and omnivorous species [9, 10]. The form of the cartilaginous or bony gill rakers, which protruded into the pharyngeal cavity, varies according to the fish's dietary preferences [11–13]. In Osteichthyes and Chondrichthyes, the gill arch pharyngeal aspect has single or paired gill rakers. In certain cases, these structures can also be found in modified form within epibranchial organs [14, 15]. Gill rakers are projections on gill arches used for food gathering and feeding habits. Bony fish utilize three filter types for food: sieve filtration, cross-flow filtration, and vortex filtration, with sieve filtration based on particle size and pore size [16].

Several literatures record the morphological features of the gills in different fish species with different feeding behaviours [12, 17–22], and it is an attractive point to be examined as it has several parts; the gill arch, gill rakers, and gill filaments that differed in their morphological characters between different fish species with different feeding behaviors and performed respiratory and digestive functions. Moreover, [23] described the cellular organization and microanatomy of gills of teleosts.

Due to the lack of knowledge *about Argyrosomus regius* gills morphology and the gills has a role related to their feeding behavior, we aim to focus on the gross and scanning electron microscopic (SEM) morphological characteristics of *Argyrosomus regius* gills in addition to the morphometric analysis of the number, length of the gill rakers and the space between them. The obtained findings will contribute to a better understanding of its ecological niche and feeding strategies in its natural environment and it will be compared with previously released information on several teleosts that exhibit various eating habits.

## Materials and methods

### Samples collection

The present work was carried out on ten mature, normal fresh *Argyrosomus regius (Asso, 1801)* fish collected in May from fish shops after catching from the Mediterranean Sea, Edku, Beheira governorate, Egypt. Their weights ranged from 250 to 300 g., and the total length was 40 to 45 cm, and they were transported in a plastic aquarium to our lab within 2 h. to examine the gills grossly and SEM. All fish were anesthetized using benzocaine (4mg/L). The samples were taken from apparently healthy *Argyrosomus regius* after veterinary examination by a special veterinarian. The collected samples followed the guidelines established for the ‘Sampling protocol for the pilot collection of catch, effort, and biological data in Egypt’ [24]. This study had been carried out with ethical permission from the Faculty of Veterinary Medicine, Alexandria University, and approved by the Institutional Animal Care and Use Committee (ALEXU-IACUC) (approval code: 171/2022).

### For gross morphological observations

The gills and oropharyngeal cavities were thoroughly washed with normal saline and examined for any injuries or abnormalities. The gill operculum was dissected, and the gills were extracted, photographed, preserved in 10% formalin solution, and transported to the dissecting laboratory [12, 13]. The gross anatomical features were photographed by a digital camera Canon (*EOS 2000-D 18–55-IS, 24.1-MP, DSLR digital camera*) in situ [21].

### For SEM observations

The gills were very attentively isolated, then small samples (about 5 cm.) of the gill arch were taken and fixed in a mixture of 2.5% paraformaldehyde and 2.5% glutaraldehyde in 0.1 M phosphate buffer for 4 hrs at 4°C. and then postfixed in osmium tetroxide 1% in phosphate buffer for 2hrs. The samples were dehydrated in increasing concentrations of ethanol before the critical point dried in carbon dioxide. Then they were sputter-coated with gold and investigated by a JEOL JSM-IT200 scanning electron microscope operating at the Faculty of Science, Alexandria University.

### For gross and morphometric analysis

The gross gill samples and images were used to count the number of gill rakers in each row from the first to the fourth gill arch (Table 1). The obtained SEM images were processed using Image J software to measure the length of the gill rakers on both the lateral and medial surfaces at the midpoint of each gill arch. Additionally, the software was used to determine the width of the spaces between the gill rakers at their base across the four gill arches, as detailed in Tables 2 and 3.

**Table 1:** Number of gill rakers on the lateral and medial surfaces of the four-gill arches of the *Argyrosomus regius*

Gill number	Rakers row	Number
**Gill rakers of the 1**^**st**^ gill arch	**Lateral**	18 ± 0.877
	**Medial**	18 ± 0. 877
**Gill rakers of the 2**^**nd**^ gill arch	**Lateral**	16 ± 0.711
	**Medial**	14 ± 0. 711
**Gill rakers of the 3**^**rd**^ gill arch	**Lateral**	14 ± 0.521
	**Medial**	12 ± 0.521
**Gill rakers of the 4**^**th**^ gill arch	**Lateral**	12 ± 0.354
	**Medial**	10 ± 0.354

**Table 2:** Length of gill rakers (µm) at the middle of each gill arch of the *Argyrosomus regius*

Gill number	Rakers row	µm
**Gill rakers of the 1**^**st**^ gill arch	**Lateral**	730.1 ± 30
	**Medial**	711.4 ± 22
**Gill rakers of the 2**^**nd**^ gill arch	**Lateral**	524 ± 23.4
	**Medial**	504 ± 18.2
**Gill rakers of the 3**^**rd**^ gill arch	**Lateral**	487 ± 13.5
	**Medial**	461.5 ± 17.1
**Gill rakers of the 4**^**th**^ gill arch	**Lateral**	390 ± 11
	**Medial**	370 ± 10

**Table 3:** Space between the bases of gill rakers at the middle of each gill arch of the of the *Argyrosomus regius*

Gill number	Rakers row	µm
**Gill rakers of the 1**^**st**^ gill arch	**Lateral**	1310.4 ± 111
	**Medial**	1023.5 ± 87
**Gill rakers of the 2**^**nd**^ gill arch	**Lateral**	1150 ± 88.4
	**Medial**	980.4 ± 71.4
**Gill rakers of the 3**^**rd**^ gill arch	**Lateral**	901 ± 68.4
	**Medial**	788.5 ± 50
**Gill rakers of the 4**^**th**^ gill arch	**Lateral**	805 ± 40
	**Medial**	520.5 ± 23.5

### Digital coloring of scanning electron microscopic images

We applied digital color enhancement to the scanning electron microscopic images using the Photo Filter 6.3.2 program to distinguish the various structures. This technique was previously illustrated by [M Elghoul, K Morsy and MMA Abumandour [25], RM Kandyel, HA El Basyouny, EE El Nahas, F Madkour, S Haddad, D Massoud, K Morsy, N Madkour and MMA Abumandour [26], MMA Abumandour, K Morsy and BG Hanafy [27], MM Abumandour, F Eldefrawy, K Morsy, N El-Bakary and BG Hanafy [28], MM Abumandour and BG Hanafy [29].

## Results

### Gross morphology

**Fig. 1:**
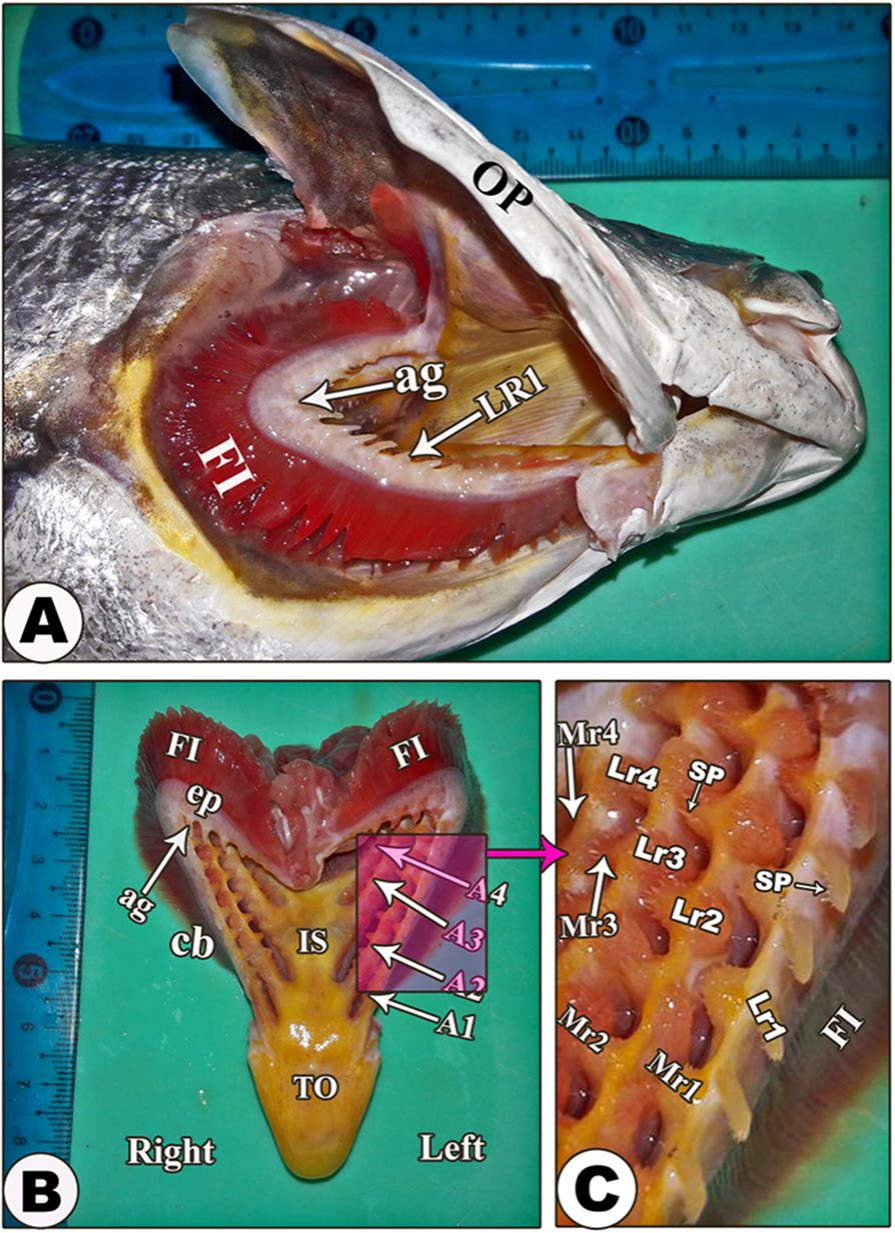
A gross anatomical photograph clarified the gills of *Argyrosomus regius*. View (**A**) shows the head region after reflection of the operculum (OP). View (**B**) shows the removed gill system. View (**C**) clarifies the magnification of the gill rakers. The 1st (A1), 2nd (A2), 3rd, (A3), and 4th (A4) gill arches; the angle (ag) between the short epibranchial (ep) and the long ceratobranchial portions (cb) of the gill arches; the gill filaments (FI); interbranchial septum (IS); the 1st (Lr1), 2nd (Lr2), 3rd, (Lr3), and 4th (Lr4) lateral rakers on the 1^st,^ 2nd, 3rd, and 4th gill arches, respectively; the 1st (Mr1), 2nd (Mr2), 3rd, (Mr3), and 4th (Mr4) medial rakers on the 1st, 2nd, 3rd, and 4th gill arches respectively; spines (SP) of the gill rakers; and tongue (TO)

The gills of *Argyrosomus regius (Asso, 1801)* were found inside two interconnected gill chambers that were bounded by the mandible ventrally, the roof of the buccopharyngeal cavity dorsally, the operculum laterally, and continued medially with each other (Fig. 1A). The gills consisted of four gill arches on each side: first (1st ), second (2nd ), third (3rd ), and fourth (4th ) from outward to inward direction (Fig. 1B). The gill arch was somewhat semilunar in shape. The gill filaments were observed on the convex border of the gill arch. The gill rakers were present on the concave border of the gill arch on its lateral and medial surfaces and carried spines (Fig. 1C).

### Gill arch

**Fig. 2:**
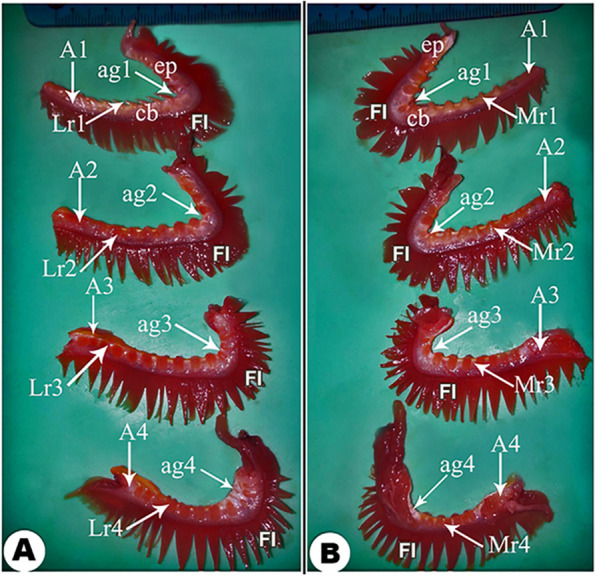
A gross anatomical photograph shows the four separated gills laterally in view (**A**) and medially in view (**B**). The 1st (A1), 2nd (A2), 3rd, (A3), and 4th (A4) gill arches; the angles (ag1, ag2, ag2, and ag4) between the short epibranchial (ep) and the long ceratobranchial portions (cb) of the 1st, 2nd, 3rd & 4th gill arches; gill filaments (FI); the 1st (Lr1), 2nd (Lr2), 3rd, (Lr3), and 4th (Lr4) lateral rakers on the 1^st,^ 2nd, 3rd, and 4th gill arches, respectively; the 1st (Mr1), 2nd (Mr2), 3rd, (Mr3), and 4th (Mr4) medial rakers on the 1st, 2nd, 3rd, and 4th gill arches, respectively.

The gill arch had two extremities: rostral and caudal. The rostral extremities were connected with each other to form the interbranchial septum between the gills of the right and left sides; this septum was yellowish in color, flattened, and smooth. The gills of the right and left sides were diverged caudolaterally, leaving a triangular area for the floor of the pharynx (Fig. 1B). The gill arches were communicated and connected to the pharynx dorsolateral wall and the operculum medial surface. All the gill arches had the same thickness and width; meanwhile, the gaps between them and their lengths were decreased toward the medial direction. The four arches were measured at 7.5 cm, 6.5 cm, 5.5 cm, and 5 cm in length, respectively. Each gill arch consisted of two portions: one long, called ceratobranchial, and one short, called epibranchial, demarcated by an angle, which appeared very narrow at the 1st gill arch and more diverged at the 4th gill arch (Fig. 2A, B/ag1 and ag4).

#### Gill rakers

The 1st gill arch had long rakers laterally and short rakers medially, while the other three gill arches had medial and lateral short rakers. The lateral rakers were dorsolaterally directed, while the medial rakers were dorsomedially directed. There was interdigitation between the rakers of the neighboring gill arches **(**Fig. 1C**).** On the 1st gill arch, the lateral rakers were pointed, triangular, and carried spines, while its medial rakers and the lateral and medial rakers on the other three gill arches were found as elevations and also carried spines (Fig. 1C**/**SP).

#### Gill filaments

Each gill filament was called a holobranch, composed of well-developed compacted medial and lateral hemibranches (Figs. 1&2**/**FI). Along the gill arches, there was a variation in the length of gill filaments; it measured about 0.7 cm in the rostral filaments, 1 cm in the middle filaments, and about 0.8 cm in the caudal filaments.

### Scanning electron microscopy (SEM)

#### Gill arch

**Fig. 3:**
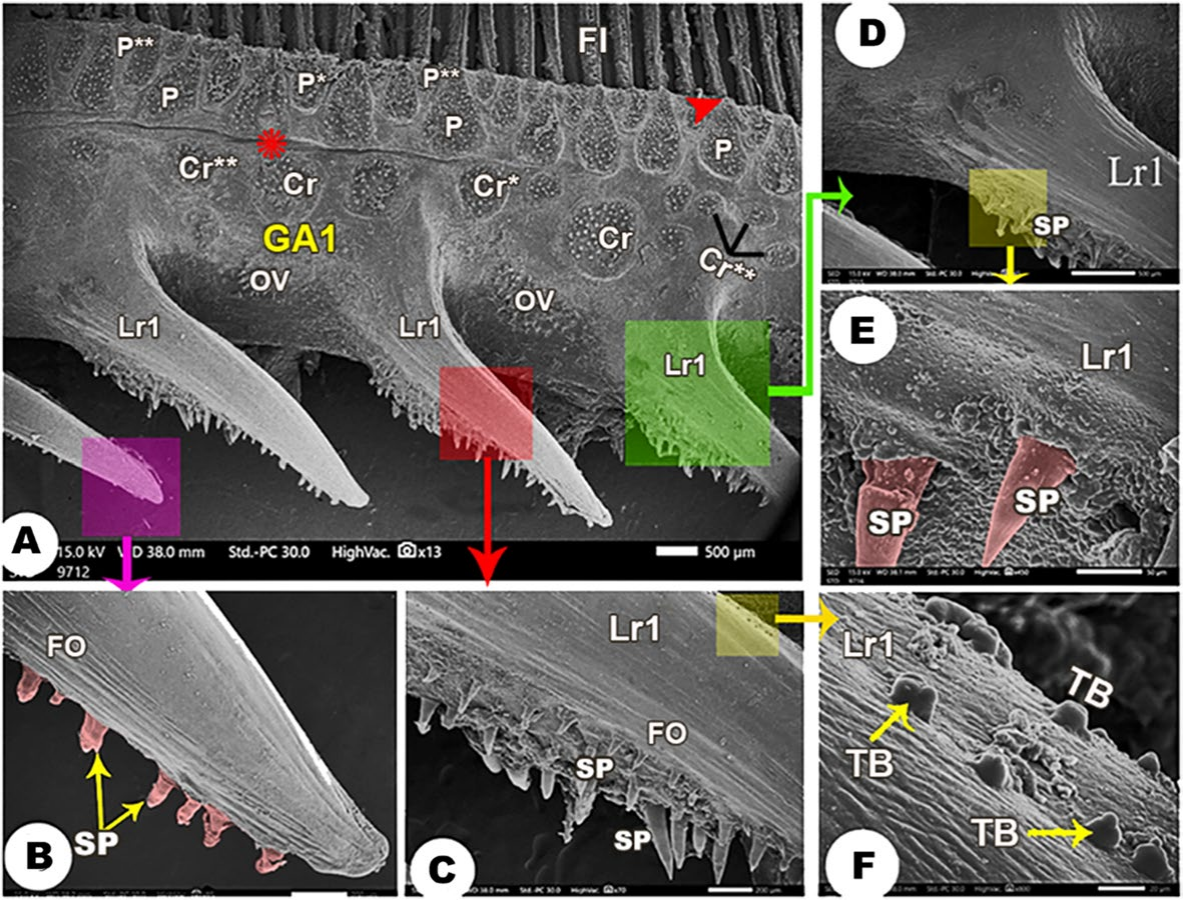
Scanning electron micrograph of the *Argyrosomus regius *shows the lateral surface of the 1st gill arch (GA1) and its rakers (Lr1) in view (**A**), the magnification of the tip of lateral rakers in view (**B**), the magnification of the middle part of the lateral rakers in view (**C**), the magnification of the origin of lateral rakers in view (**D**), the magnification of spines (SP) presented on the lateral rakers in view (**E**), and the taste buds (TB) of the lateral rakers in view (**F**). The large (Cr), moderate (Cr*), and small (Cr**) circular groups of spines (SP); gill filaments (FI), folds (FO), the large (P), moderate (P*), and small (P**) pear-shaped groups, and the oval group (OV) of spines. The epithelial flap between the gill filaments (red arrowhead) and the gill arch and the wrinkling of the epithelium (asterisk)

**Fig. 4:**
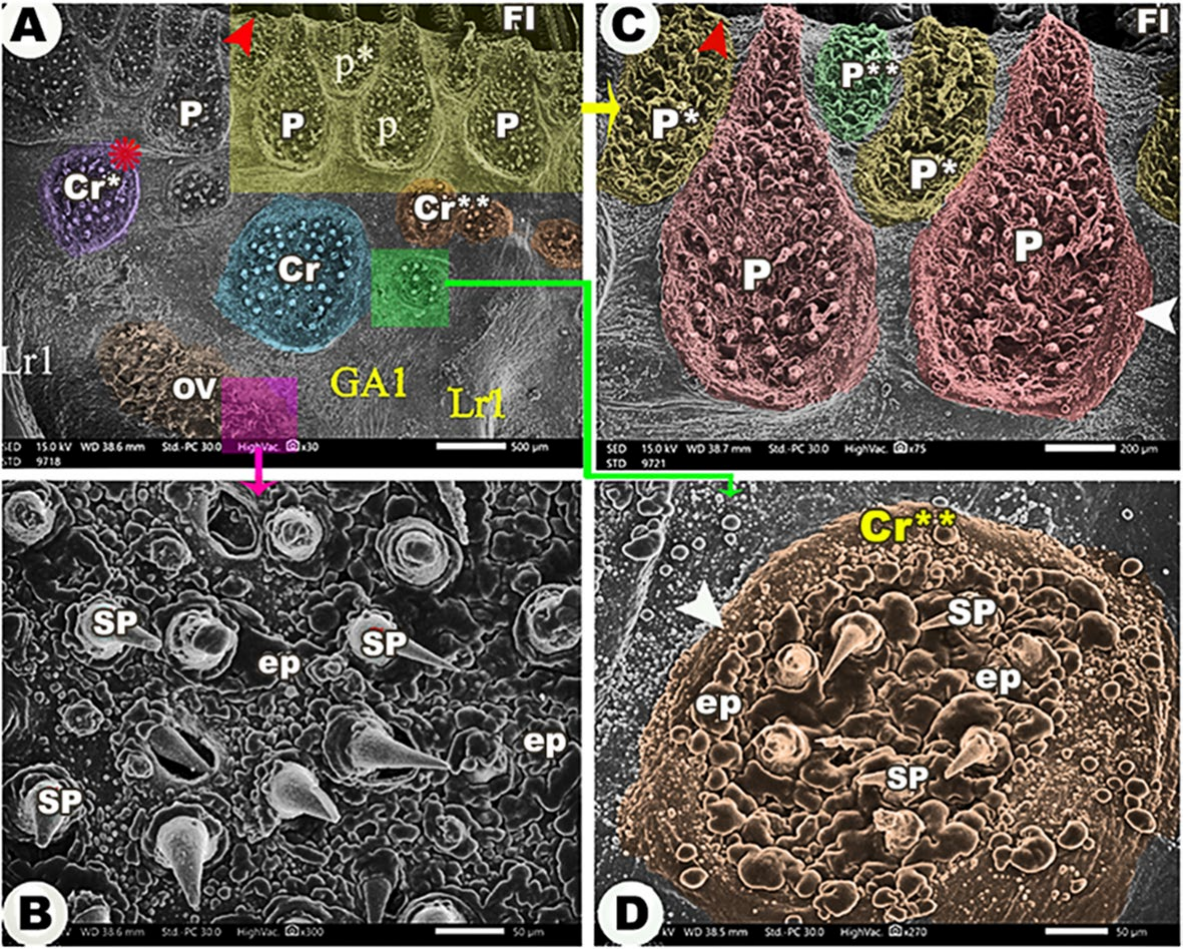
Scanning electron micrograph shows the large (P), moderate (P*), and small (P**) pear-shaped groups of spines (SP) on the lateral surface of the 1st gill arch (GA1), the large (Cr), moderate (Cr*), and small circular (Cr**) groups of spines, and the oval group (OV) of spines in view (**A**), the magnification of the spines in view (**B**), and the magnification of pear-shaped groups in view (**C**) and circular groups in view (**D**). The epithelial protrusions (ep), gill filaments (FI), lateral rakers on the 1st gill arch (Lr1), the epithelial flap between the gill filaments and the gill arch (red arrowheads), the elevated contour of the pear-shaped and circular groups of spines (white arrowheads), and the wrinkling of the epithelium (asterisk)

**Fig. 5:**
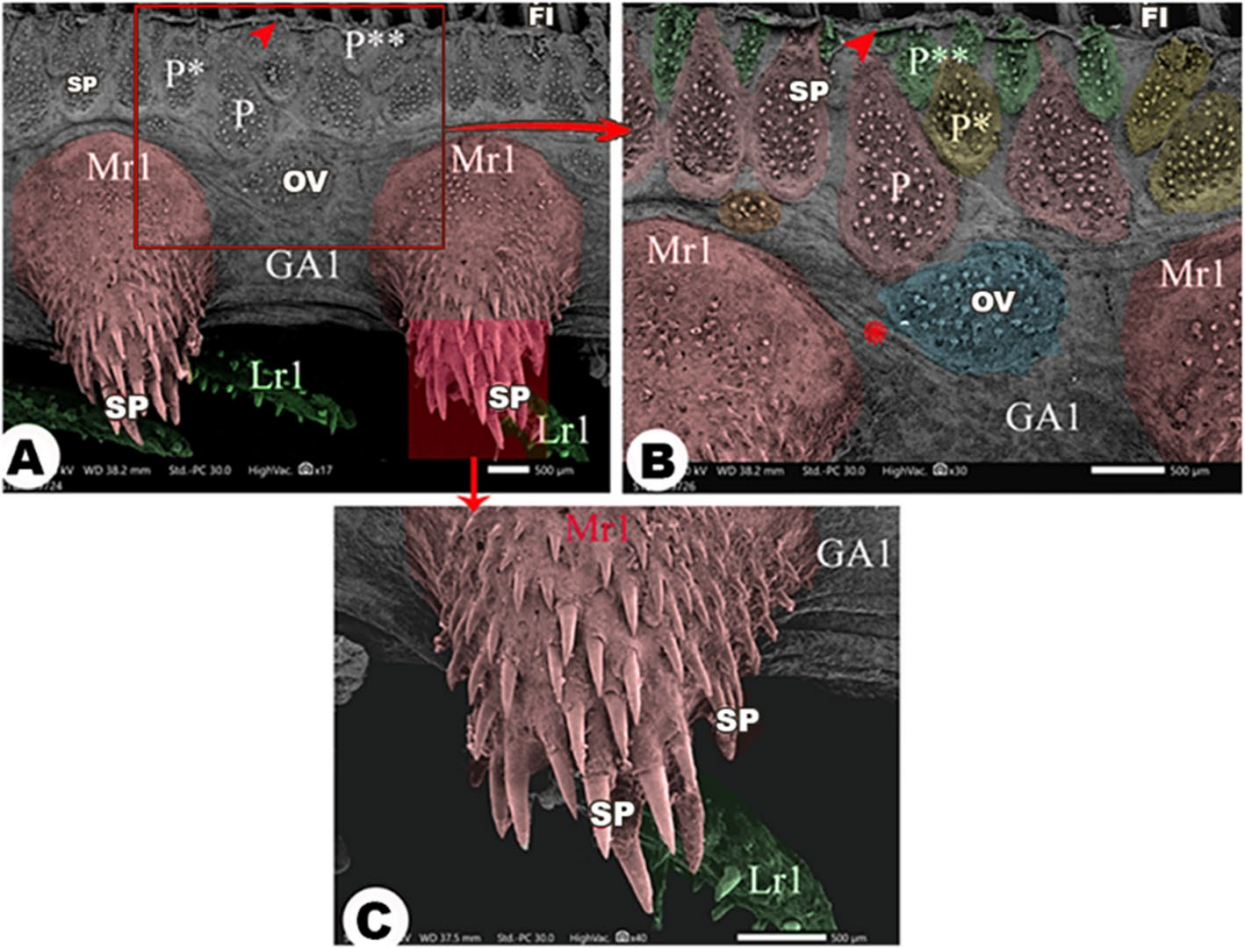
Scanning electron micrograph shows the medial surface of the 1st gill arch (GA1) and its rakers (Mr1), the large (P), moderate (P*) and small (P**) pear shaped groups and the oval group (OV) of spines (SP) in view (**A**-**B**) and the magnification of Mr1 in view (**C**). The gill filaments (FI), the epithelial flap between the gill filaments and the gill arch (red arrowheads), and the wrinkling of the epithelium (asterisk)

**Fig. 6:**
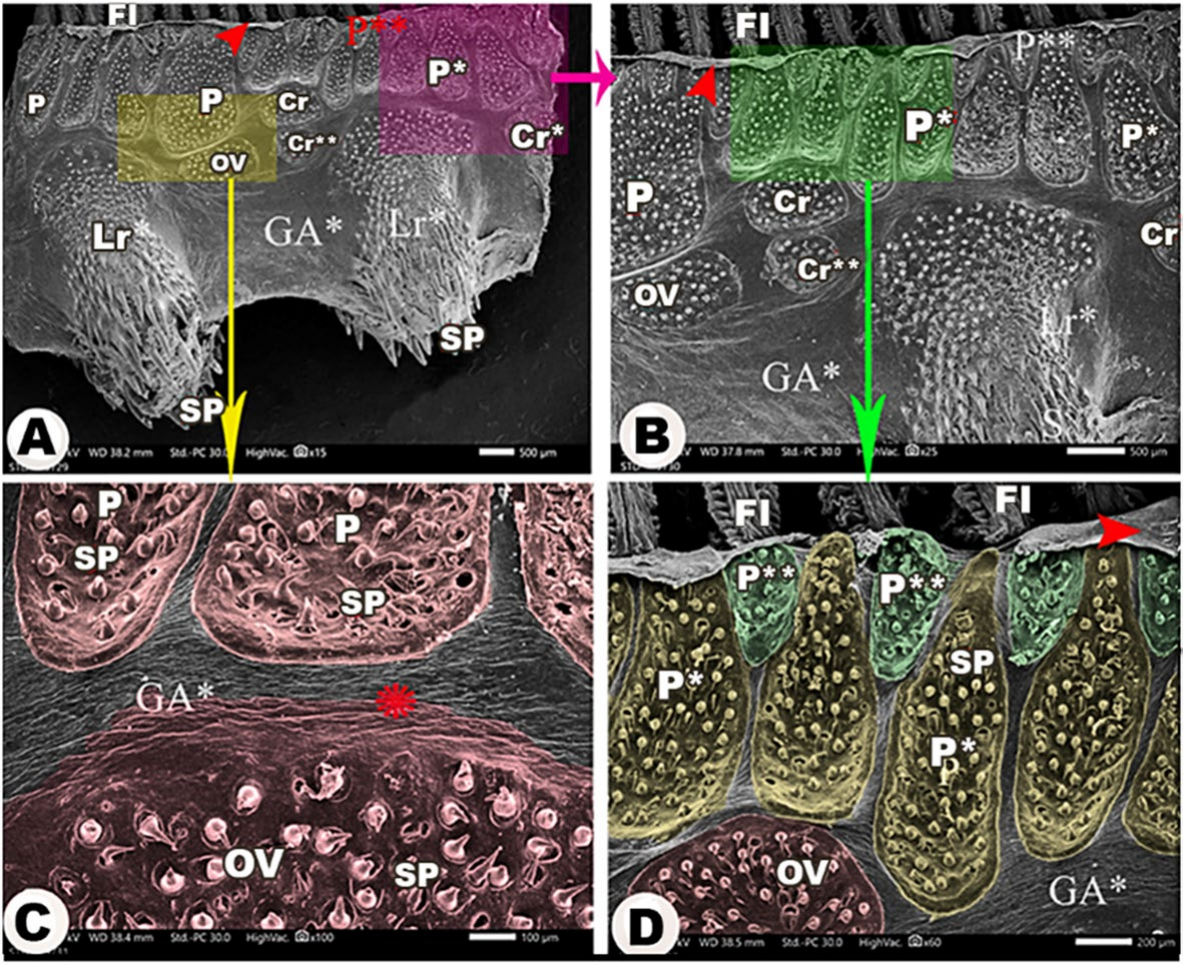
Scanning electron micrograph shows the lateral surface of the other three gill arches (GA*) and its rakers (Lr*), the large (P), moderate (P*), and small (P**) pear-shaped groups of spines (SP), the large (Cr), moderate (Cr*), and small circular (Cr**) groups of spines, and the oval group (OV) of spines in view (**A**-**B**) and the magnification of their spines in view (**C**-**D**). The gill filaments (FI), the epithelial flap between the gill filaments and the gill arch (red arrowheads), and the wrinkling of the epithelium (asterisk)

**Fig. 7:**
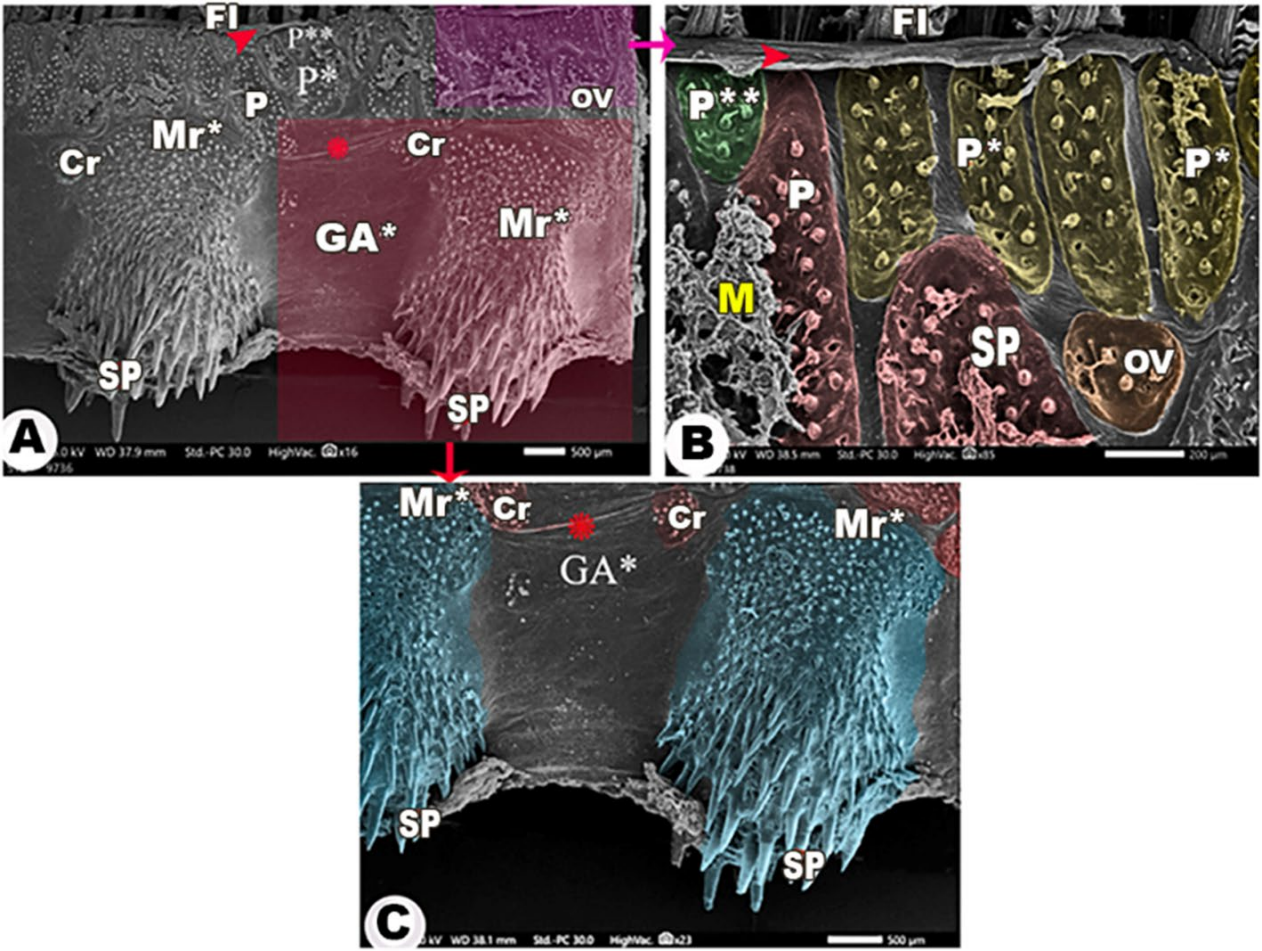
Scanning electron micrograph shows the medial surface of the other three gill arches (GA*) and its rakers (Mr*), the large (P), moderate (P*), and small (P**) pear-shaped groups, oval (OV), and circular (Cr) groups of spines (SP) in view (**A**), the magnification of the pear-shaped groups of spines in view (**B**), and the magnification of Mr* in view (**C**). The gill filaments (FI), mucous (M), the epithelial flap between the gill filaments and the gill arch (red arrowheads), and the wrinkling of the epithelium (asterisk)

The medial and lateral surfaces of the four gill arches were wrinkled (Figs. 3A, 4A, 5B, 6C & 7A). There was an epithelial flap between the gill arch, and the filaments appeared medially and laterally **(**Figs. 3A, 4, 6&7A,B**)**.

Laterally, all gill arches carried large, moderate, and small pear-shaped groups of spines near the convex border that carried the gill filaments (Figs. 3A, 4A, 6A,B& 6D). Below these pear-shaped groups and near the origins of the gill rakers, large, moderate, and small circular groups of spines were observed **(**Figs. 3A, 4A, & 6A, B), in addition to the presence of oval-shaped groups of spines **(**Figs. 3A, 4A & 6A,B**/**OV).

The medial surface of the 1st gill arch carried only large, moderate, and small pear-shaped groups of spines (Fig. 5A,B) and oval groups of spines **(**Fig. 5A,B/OV) in the same position as the lateral surface of the arch. These groups of spines had elevated contours **(**Fig. 4C,D**)**, and the spines were curved and pointed with epithelial protrusions between them **(**Fig. 4B,D). On the other hand, the other three gill arches mostly had the same-shaped groups of spines, but the oval groups of spines were found between the pear-shaped groups, and only small circular groups were found near the origins of gill rakers **(**Fig. 7A-C).

#### Gill rakers

The lateral rakers of the 1st gill arch were long with rounded ends, and they had longitudinal folds on their surface. Only one border of these rakers carried spines with pointed ends (Fig. 3A,E/Lr1). The spines were abundant at the origin and the middle part of these rakers (Fig. 3C,D/SP), and they were few in number at the tips of these rakers (Fig. 3B/SP). The taste buds were observed on the middle part of these rakers on the border that was devoid of the spines (Fig. 3/TB).While the medial rakers of the 1st gill arch were triangular in outline (Fig. 5A,C/Mr1) and carried pointed spines that increased in their length toward the tip of these rakers (Fig. 5A,C/SP).

The rakers of the other three gill arches had the appearance of flask laterally and medially (Figs. 6A/Lr* & 7A,C/Mr*) and carried pointed spines, increasing in their length towards the rakers tips (Figs. 6A & 7A,C/SP).

#### Gill filaments

**Fig. 8:**
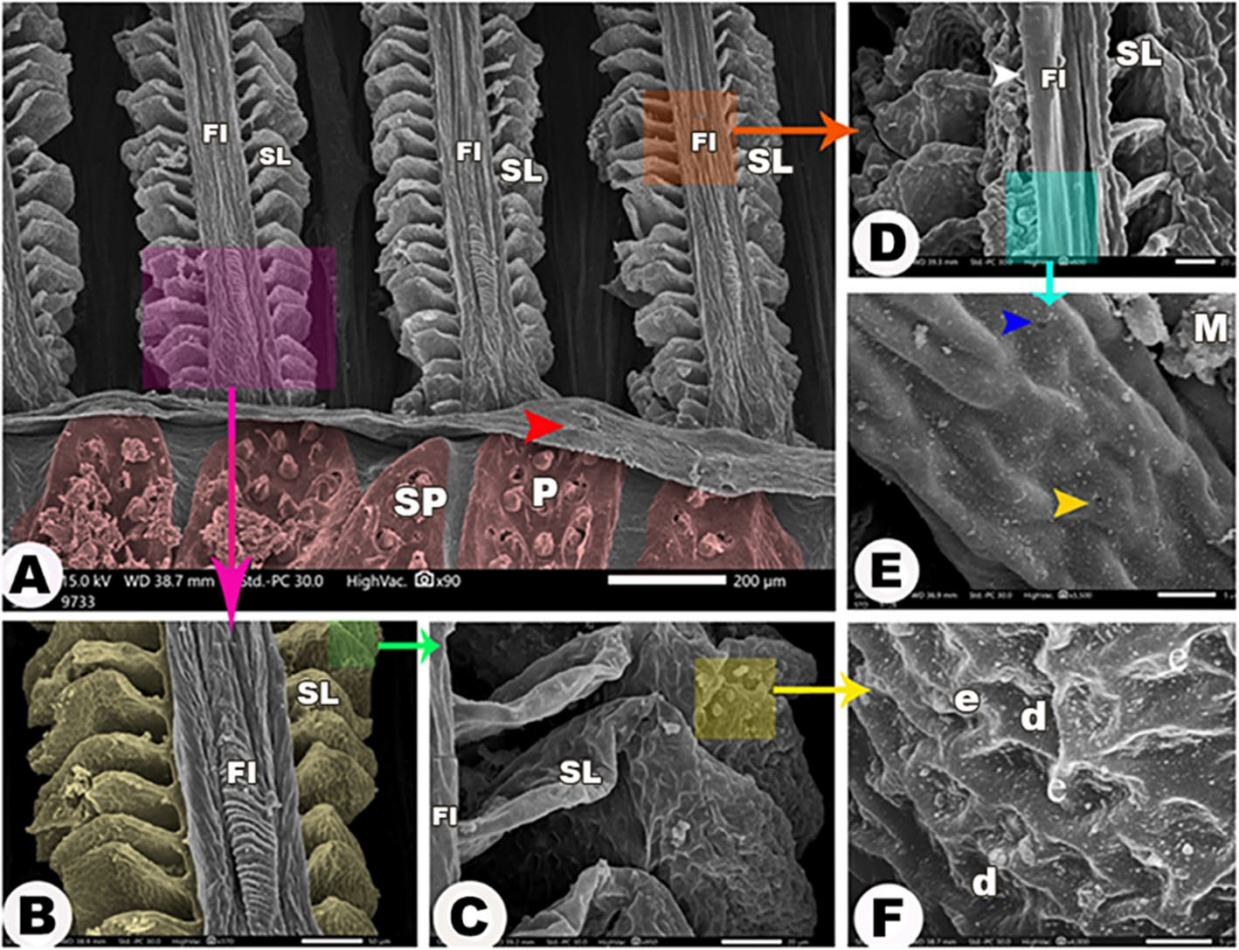
Scanning electron micrograph shows the primary gill filaments (FI) carried secondary lamellae (SL) in view (**A**), the magnification of the primary gill filaments in views (**B**, **D**, and **E**), and the magnification of the secondary lamellae in views (**C** and **F**). The elevated areas (e), depressed area (d), mucous (M), and pear-shaped (P) groups of spines (SP). The gill filaments (FI), the epithelial flap between the gill filaments and the gill arch (red arrowheads), the wrinkling of the epithelium (asterisk), the chloride cells (yellow arrowhead), the openings of the mucous cells (blue arrowhead), and the tubercles on certain parts of the primary gill filaments (white arrowhead).

The primary gill filaments had secondary lamellae on their sides (Fig. 8A). The primary gill filaments had folds and tubercles on their surface (Fig. 8D); some folds gave the appearance of a helix on the surface of the parts that were observed near the gill arch convex border (Fig. 8B), and they carried orifices of mucous cells and pores of chloride cells without the presence of distinctive structures inside them (Fig. 8E). Elevated and depressed areas were observed on the secondary lamellae surfaces, which were characterized by the presence of (Fig. 8F), and their free ends were thicker than their attached ends(Fig. 8C).

### Morphometric analysis

The average numbers of the lateral rakers were 18, 16, 14, and 12 on the 1st, 2nd, 3rd, and 4th gill arches, respectively. While the average numbers of the medial rakers were 18, 14, 12, and 10 on the 1st, 2nd, 3rd, and 4th gill arches, respectively (Table 1).

The length of the gill rakers was decreased medialward (Table 2). The length of the gill rakers on the 1st gill arch was the longest on each lateral and medial surfaces reached 730.1 ± 30 and 711.4 ± 22 µm of the lateral and medial, respectively. Meanwhile, the shortest ones were seen on the 4th gill arch that reached 390 ± 11 and 370 ± 10 µm of the lateral and medial, respectively.

The space between the bases of gill rakers at the middle of each gill arch was wider between them on each lateral and medial surface of the 1st gill arch that reached 1310.4 ± 111 and 1023.5 ± 87 µm of the lateral and medial, respectively. Meanwhile, the narrowest space was observed between them on the 4th gill arch that reached 805 ± 40 and 520.5 ± 23.5 µm of the lateral and medial, respectively (Table 3).

## Discussion

Our study provided a comprehensive morphological description of *Argyrosomus regius (Asso, 1801)* gills through the gross, scanning electron microscopic examinations and morphometric analysis of the number, length of the gill rakers and the space between them. We revealed that *Argyrosomus regius* gill rakers poss unique structural specifications related to its feeding behavior. Our findings will contribute to a better understanding of the ecological niche and feeding strategies of *Argyrosomus regius* in its natural environment.

*Argyrosomus regius* gills were located inside gill chambers that bounded dorsally by the oropharyngeal cavity roof, the mandible ventrally, the operculum laterally, and connected medially with each other. These finsings were observed in Grey Gurnard and Striped Red Mullet by M Abumandour and NE El-Bakary [13], in European hake by BG Hanafy [20], in Bagrus bayad by NF Bassuoni [19], and in European barracuda by BG Hanafy [21]. While M Abumandour and MS Gewaily [17] in puffer fish recorded that the gill chambers were bounded by three horizontal rods and the mandible ventrally, by the roof of the oral cavity dorsally, by the base of the pectoral fin caudally, and by three layered gills covering laterally and appearing continuous with each other medially.

*Argyrosomus regius* had a single operculum covering the gills and left a ventrolateral opening. These findings are similar to those reported in most teleosts [13, 20, 21, 30–32]. Meanwhile, in puffer fish, MAbumandour and MS Gewaily [17] reported three layers of gill covering. Additionally, in the rays and sharks, individual gill openings were observed [33].

*Argyrosomus regius* are carnivorous fish feed on decapoda, teleosts and mysidacea [34, 35]. So, their gill arches are characterized by presence of marked angles between their ceratobranchial and epibranchial portions. These results agree with those described by U Kumari, M Yashpal, S Mittal and AK Mittal [36] in catfish *Rita rita*, BG Hanafy [20] in European hake, NF Bassuoni [19] in Bagrus bayad and BG Hanafy [21] in European barracuda. Meanwhile, FE Hossler, JR Ruby and TD McIlwain [37] in filter feeding mullets Mugil cephalus and M Abumandour and NE El-Bakary [13] in the Striped mullet fish and Grey gurnard fish didn't observe this angle.

According to the most recent research, *Argyrosomus regius's* gill system was made up of four pairs of gill arches: the first, second, third, and fourth gill arches, arranged laterally to the medially. These gill arches were somewhat crescentic; the gill filaments were observed on its convex border and the gill rakers were present on its concave border like most bony fish [4, 6, 7, 19–21, 38–42]. Whereas, M Abumandour and MS Gewaily [17] in puffer fish and M Abumandour and NE El-Bakary [13] in striped red mullet fish recorded the presence of three gill arches on each side of the gill chamber. Moreover in catfish, U Kumari, M Yashpal, S Mittal and AK Mittal [36] showed the presence of five gill arches on each side of the gill chamber, but the fifth one didn't have gill filaments and appeared ill-developed. In contrast, J Arellano, V Storch and C Sarasquete [5] reported that the fifth pair that present in Senegal sole was well-developed with gill filaments. Additionally, the cartilaginous fish had 5–7 pairs and the primitive jawless fish had seven gill arches on each side of the gill chamber [43].

*Argyrosomus regius* gill arches carried medial and lateral rakers. The observation of the rakers with a varied appearance in different fish with different feeding habits was previously recorded by, E Elsheikh [32]; H Khalaf-Allah, A Azab and A Mohamed [44]; M Abumandour and NE El-Bakary [13]; T Märss, MV Wilson, T Saat and H Špilev [45]; BG Hanafy [20] and BG Hanafy [21]. On the other hand, NF Bassuoni [19] observed the rakers on the third and fourth arch laterally and medially, while the first and second arch only had lateral rakers. The morphology and functions of gill rakers in different fish species are influenced by their feeding habits and habitat. The long rakers are abundant in the filter feeding species, while the short rakers are recorded in few numbers in carnivorous and omnivorous fish [9, 10].

The data from earlier publications indicated that the fish which consume large prey contained a small number of gill rakers. According to CE Bond [46], plankton feeders had garnish gill rakers, which were long, variable, and numerous lamellae that acted as a sieve, catching solid food while carrying water. JR Mummert and RW Drenner [10] noted that gill rakers may alter as a fish grows. Similarly, M McCormick [47] noted that the ontogeny of Cheilodactylus spectabilis diet was shifted as a result of the interaction between feeding mechanics, microhabitat selection, and growth. In this scenario, both juveniles and adults choose specific taxa from the available turf microfauna, with juveniles consuming smaller sizes to feed. The size, shape, and space between gill rakers were likely indicative of the feeding patterns of the fish species. Fish with short rakers are carnivores and omnivores, while those with many long rakers are filter feeders [12, 13, 19, 20, 48–50].

*Argyrosomus regius* gill rakers carried a lot of spines that prevent smooth, slimy, and slippery captured preys from escaping and help in seizing them [51, 52]. We revealed that there were spine systems on the gill arches, in which the four gill arche's lateral surfaces contained pear-shaped, circular, and oval-shaped spine groups with varying sizes, while the medial surface of the 1st gill arch only had different sizes of pear-shaped and oval groups of spines, but the other gill arches had the same-shaped groups of spines beside the presence of small circular groups, while the lateral rakers of the 1st gill arch showed long, and only one border carried pointed spines, while its medial rakers were triangular. Functionally, the lateral gill rakers of carnivorous fishes of the 1st gill arch carried minute spines that prevented the escape of the slippery, slimy, and smooth prey, while the rakers on the subsequent rows were shorter [13, 48, 51, 52]. The present work revealed the presence of spines arranged in different forms; pear-shaped, circular, and oval groups with different sizes; large, moderate, and small medially and laterally on the gill arches and gill rakers to adapt the *Argyrosomus regius* fish to its feeding ecology and food preferences [13, 36].

The highest number of the *Argyrosomus regius* gill rakers was on the 1st gill arch, then the raker numbers decreased medailward. The increasing number of gill rakers enhances cross-flow filtering, while narrowly spaced gill rakers restrict the escape possibilities of small prey [53–56]. The strong correlation between the gill raker gap and standard length influences the prey size relationship [47]. In our work, the taste buds were found on the middle part of rakers that presented laterally on the first arch on the border that is devoid of the spines to help in sorting of food particles in the pharynx before reaching to the esophagus. In the gills, the taste buds are correlated to tasting of the food in the pharyngeal cavity [57, 58]. L Fishelson, Y Delarea and A Zverdling [57] recorded the presence of the taste buds on the basal parts of the gill arches and skin and in the oropharyngeal cavity. The presence of the taste buds with the sharp mouth teeth in this carnivorous fish is functioning for the occurrence of gustation and food processing simultaneously in the pharynx, and it may help the fish to determine the most suitable particles of the food to swallow them as suggested by PJ Linser, WE Carr, HS Cate, CD Derby and JC Netherton III [59] in micropterus salmoides.

The surface epithelium of the gill arches in *Argyrosomus regius* appeared wrinkled to increase the area for trapping, holding, and distribution of mucous and to provide stretching area. These observations coincide with those clarified by MM Abumandour [18] and M Abumandour and NE El-Bakary [13]. Likewise, U Kumari, M Yashpal, S Mittal and AK Mittal [36] proposed that these micro-ridges provide mechanical flexibility to increase the surface firmness of arches and rakers. The ejection of mucous from the openings of the mucous cells on the arches surfaces helped in lubrication and the smooth passage of the food items to prevent mechanical injuries of the epithelium by the captured preys [60–63], and the mucous provides immunological responses in case of the infection of the fish [64, 65].

In the present work, the chloride cell pores were observed on the primary gill filament surfaces without any characteristic composition inside them to help in osmoregulation. These findings agree with M Abumandour and MS Gewaily [17] in puffer fish, BG Hanafy [20] in European hake, NF Bassuoni [19] in Bagrus bayad, and BG Hanafy [21] in European barracuda. The chloride cells of freshwater fish species have a wide apex with less obvious crypts and are devoid of pits [30, 39, 66]. Furthermore, [R Carmona, M García-Gallego, A Sanz, A Domezain and M Ostos‐Garrido [67] reported that the chloride cells of seawater fish species were distinguished by the presence of shallow crypts or depressions at the chloride cell pole and deep pits with or without visible apical extension, and they increased in number and size over most of the surface of lamellae.

## Conclusion

Considering that the *Argyrosomus regius (Asso, 1801)* is a good candidate in aquaculture, we gave a concern to the morphological characters of their gills as they had a role related to their feeding behavior. Laterally, the four arches had spines in different forms: pear-shaped and circular groups with different sizes in addition to oval groups. While the 1st gill arch only had different sizes of pear-shaped groups and oval groups medially. The other three gill arches had the same-shaped groups of spines in addition to small circular groups medially. The 1st gill arch lateral rakers were long, and only one border carried spines with pointed ends. These spines prevent the particles of the food from passing to the gill filaments and prevent the suffocation of the fish. Finally, we recommend further investigation of this fish's gills using various anatomical techniques such as histological and immunofluorescence microscopy to comprehend their adaptations to aquatic environments.

## Data Availability

The datasets used and/or analyzed during the current study are available from the corresponding author on reasonable request.
